# Supplementary material for: Surface Adsorption at the Thermodynamic Limit Using Periodic DLPNO-MP2 Theory: A Study of CO on MgO at Dilute and Dense Coverages
Source: J Chem Theory Comput. 2026 Apr 13;22(8):3927–36. doi: 10.1021/acs.jctc.5c02179 (PMC13130862; doi:10.1021/acs.jctc.5c02179)
Supplement: Supplementary file 1 [file ct5c02179_si_002.pdf]

Supplementary Material:

Surface adsorption at the thermodynamic limit using periodic DLPNO-MP2 theory: A study of CO on MgO at dilute and dense coverages

Andrew Zhu, Poramas Komonvasee, Arman Nejad, and David P. Tew*

University of Oxford, South Parks Road, Oxford, OX1 3QZ, UK. E-mail: david.tew@chem.ox.ac.uk

S1 Unit cell geometries

The geometries and cell parameters are reported in the Turbomole format

```
$cell
  a b c  α   β   γ
```

where all lengths are in Bohr and angles in degree.

Dilute Coverage Regime

The geometries in this section are taken from the work by Ye and Berkelbach[1].

1 CO on 2 × 2 MgO surface ($\Theta = 0.25$)

```
$cell
  11.285742096   11.285742096  90
$coord
       2.8214488818          2.8214134306         10.6032541411       c
       2.8215257748          2.8214088385         12.7588060365       o
       0.0000000000          0.0000000000          1.8897261246       mg
       0.0000000000          5.6428714587          1.8897261246       mg
       5.6428714587          0.0000000000          1.8897261246       mg
       5.6428714587          5.6428714587          1.8897261246       mg
       2.8214008261          2.8214335562          5.9545282092       mg
       2.8214754136          8.4642984197          5.8611450306       mg
       8.4642871570          2.8214467842          5.8611510022       mg
       8.4643301293          8.4642878751          5.8640625032       mg
       2.8214348034          2.8214348034          1.8897261246       o
       2.8214348034          8.4643062621          1.8897261246       o
       8.4643062621          2.8214348034          1.8897261246       o
       8.4643062621          8.4643062621          1.8897261246       o
      -0.0128432968         -0.0128456968          5.9643583000       o
      -0.0127778178          5.6556489364          5.9651761167       o
       5.6556236329         -0.0127849610          5.9651417426       o
       5.6557114674          5.6556888285          5.9643673518       o
```

1 CO on 3 × 3 MgO surface ($\Theta = \frac{1}{9}$)

```
$cell
16.928613144   16.928613144  90
```

```
$coord
    2.82143947100866      2.82143603170712     10.5932477200399        c
    2.82143408528921      2.82143569155641     12.7487996155071        o
    0.00000000000000      0.00000000000000      1.88972612462577      mg
   11.28574291740374     11.28574291740374      1.88972612462577      mg
   11.28574291740374      5.64287145870187      1.88972612462577      mg
   11.28574291740374      0.00000000000000      1.88972612462577      mg
    5.64287145870187     11.28574291740374      1.88972612462577      mg
    5.64287145870187      5.64287145870187      1.88972612462577      mg
    5.64287145870187      0.00000000000000      1.88972612462577      mg
    0.00000000000000      5.64287145870187      1.88972612462577      mg
    0.00000000000000     11.28574291740374      1.88972612462577      mg
    2.82142628072031     14.10633049987543      5.87026265681236      mg
    2.82145848165348      2.82142972002186      5.94452178813993      mg
   14.10580923782121      8.46566626018438      5.87454679821772      mg
   14.10630833338799      2.82144534805691      5.87031127946555      mg
    2.82141044481539      8.46515006259618      5.87026203320274      mg
    8.46566004298543      8.46566801762967      5.87455159812207      mg
   14.10580931341026     14.10581426449270      5.87458729504857      mg
    8.46519772148904      2.82142473114489      5.87031214873957      mg
    8.46567294981486     14.10579508377254      5.87456881352707      mg
   14.10717772078888     14.10717772078888      1.88972612462577       o
   14.10717772078888      8.46430626208701      1.88972612462577       o
   14.10717772078888      2.82143480338514      1.88972612462577       o
    8.46430626208701     14.10717772078888      1.88972612462577       o
    8.46430626208701      2.82143480338514      1.88972612462577       o
    8.46430626208701      8.46430626208701      1.88972612462577       o
    2.82143480338514      2.82143480338514      1.88972612462577       o
    2.82143480338514     14.10717772078888      1.88972612462577       o
    2.82143480338514      8.46430626208701      1.88972612462577       o
    5.64146945199275     11.28573879780079      5.97146467174535       o
    0.00138955341396     11.28574236938317      5.97146881024556       o
   -0.01728900982789      5.66014661683727      5.95918965334666       o
    5.66014217598088      5.66013616665180      5.95921051592307       o
   11.28574781179441      0.00139898314732      5.97149403808932       o
   -0.01728005252606     -0.01727003697760      5.95921187652588       o
   11.28573961038303      5.64147226768468      5.97148557211628       o
    5.66016054411881     -0.01728058164938      5.95919178873718       o
   11.28574278512292     11.28574040406800      5.96921907239693       o
```

1 CO on 4 × 4 MgO surface ($\Theta = \frac{1}{16}$)

```
$cell
22.571484192 22.571484192  90
$coord
    2.82144204103619      2.82144283472116     10.6034772988813        c
    2.82145479668753      2.82143809150859     12.7588845925054        o
    0.00000000000000      0.00000000000000      1.88972612462577      mg
   16.92861435720836     11.28574291740374      1.88972612462577      mg
    5.64287145870187     11.28574291740374      1.88972612462577      mg
    5.64287145870187     16.92861435720836      1.88972612462577      mg
   11.28574291740374      0.00000000000000      1.88972612462577      mg
   11.28574291740374      5.64287145870187      1.88972612462577      mg
   11.28574291740374     11.28574291740374      1.88972612462577      mg
   16.92861435720836      5.64287145870187      1.88972612462577      mg
   11.28574291740374     16.92861435720836      1.88972612462577      mg
   16.92861435720836      0.00000000000000      1.88972612462577      mg
    5.64287145870187      5.64287145870187      1.88972612462577      mg
   16.92861435720836     16.92861435720836      1.88972612462577      mg
```

```
    0.00000000000000     16.92861435720836      1.88972612462577       mg
    0.00000000000000      5.64287145870187      1.88972612462577       mg
    0.00000000000000     11.28574291740374      1.88972612462577       mg
    5.64287145870187      0.00000000000000      1.88972612462577       mg
    2.82142597836413      2.82143181761786      5.95475136698132       mg
   14.10717928926156     19.75085136823066      5.87976684538355       mg
   14.10717775858340     14.10717758850805      5.87944808638085       mg
    2.82142306818590      8.46820909455494      5.86880201190159       mg
   14.10717711607652      8.46350173008671      5.87976625956846       mg
    8.46816965597072      2.82143074047397      5.86879371600391       mg
   19.74704077329487      8.46730510626870      5.87627321421065       mg
    2.82143661752221     14.10717656805595      5.88008698388633       mg
   19.74705704383680     19.74706511296736      5.87626338763480       mg
    8.46731264627594     19.74705305651468      5.87627081425847       mg
    8.46350781500483     14.10717936485061      5.87976699656164       mg
    2.82144136073479     19.74614211403631      5.86880598032645       mg
   19.75084685178522     14.10717792865876      5.87977071932211       mg
    8.46729834104917      8.46730357559054      5.87626478603214       mg
   14.10718083883699      2.82143476559061      5.88009159481807       mg
   19.74617340790093      2.82144462996098      5.86878638386654       mg
   19.75004916059349      2.82143480338514      1.88972612462577        o
    8.46430626208701     19.75004916059349      1.88972612462577        o
   14.10717772078888      2.82143480338514      1.88972612462577        o
    8.46430626208701      8.46430626208701      1.88972612462577        o
   19.75004916059349      8.46430626208701      1.88972612462577        o
   14.10717772078888     14.10717772078888      1.88972612462577        o
   14.10717772078888     19.75004916059349      1.88972612462577        o
   14.10717772078888      8.46430626208701      1.88972612462577        o
    8.46430626208701     14.10717772078888      1.88972612462577        o
    2.82143480338514     19.75004916059349      1.88972612462577        o
   19.75004916059349     14.10717772078888      1.88972612462577        o
    2.82143480338514      8.46430626208701      1.88972612462577        o
    2.82143480338514     14.10717772078888      1.88972612462577        o
    2.82143480338514      2.82143480338514      1.88972612462577        o
    8.46430626208701      2.82143480338514      1.88972612462577        o
   19.75004916059349     19.75004916059349      1.88972612462577        o
   16.92821176995477     11.28614093152012      5.97052243540235        o
   16.92645859543991      5.64229084035008      5.97167673681105        o
   16.92646305519356      0.00058313168754      5.97167409119448        o
    5.64229652842572     16.92645549628906      5.97167395891365        o
   11.28614183858866     11.28614285904076      5.97051612371709        o
   11.28789359580892      5.64229084035008      5.97166948026273        o
   11.28789660047346      0.00057880421471      5.97166929129012        o
    5.64228980100071     11.28790072007641      5.97167448803696        o
    5.65983263884167      5.65984760547257      5.96179482978207        o
    5.65984297564357     -0.01698243955870      5.96180388157021        o
    0.00057831288592     16.92645825528920      5.97167547069455        o
    0.00057555388578     11.28790036102845      5.97167796513303        o
   -0.01698468833279      5.65985584467848      5.96178908501465        o
   -0.01697070435946     -0.01696960831831      5.96179919504942        o
   11.28614478656141     16.92821424549599      5.97051901499806        o
   16.92821396203707     16.92821634309199      5.97051721975824        o
```

Dense Coverage Regime

The geometries in this section are CO molecules adsorbed on a pristine MgO surface without geometry optimization.

1 CO on 2 × 2 MgO surface ($\Theta = 0.25$)

```
$cell
11.285742096   11.285742096  90
$coord
 -8.4643072924      14.1071768992       7.12154955      c
 -8.4643072924      14.1071768992       9.277101446     o
-14.1071783403      14.1071768992       2.4728236184    mg
-14.1071783403      19.7500479471       2.4728236184    mg
-11.2857420958      11.2857420958      -1.5186979106    mg
 -8.4643072924      14.1071768992       2.4728236184    mg
-11.2857420958      16.9286131437      -1.5186979106    mg
 -8.4643072924      19.7500479471       2.4728236184    mg
 -5.6428710479      11.2857420958      -1.5186979106    mg
 -5.6428710479      16.9286131437      -1.5186979106    mg
-14.1071783403      14.1071768992      -1.5186979106     o
-14.1071783403      19.7500479471      -1.5186979106     o
 -8.4643072924      14.1071768992      -1.5186979106     o
-11.2857420958      11.2857420958       2.5645722028     o
 -8.4643072924      19.7500479471      -1.5186979106     o
-11.2857420958      16.9286131437       2.5645722028     o
 -5.6428710479      11.2857420958       2.5645722028     o
 -5.6428710479      16.9286131437       2.5645722028     o
```

2 CO on 2 × 2 MgO surface ($\Theta = 0.50$)

```
$cell
11.285742096   11.285742096  90
$coord
-14.1071783403      19.7500479471       7.12154955      c
 -8.4643072924      14.1071768992       7.12154955      c
-14.1071783403      19.7500479471       9.277101446     o
 -8.4643072924      14.1071768992       9.277101446     o
-14.1071783403      14.1071768992       2.4728236184    mg
-14.1071783403      19.7500479471       2.4728236184    mg
-11.2857420958      11.2857420958      -1.5186979106    mg
 -8.4643072924      14.1071768992       2.4728236184    mg
-11.2857420958      16.9286131437      -1.5186979106    mg
 -8.4643072924      19.7500479471       2.4728236184    mg
 -5.6428710479      11.2857420958      -1.5186979106    mg
 -5.6428710479      16.9286131437      -1.5186979106    mg
-14.1071783403      14.1071768992      -1.5186979106     o
-14.1071783403      19.7500479471      -1.5186979106     o
 -8.4643072924      14.1071768992      -1.5186979106     o
-11.2857420958      11.2857420958       2.5645722028     o
 -8.4643072924      19.7500479471      -1.5186979106     o
-11.2857420958      16.9286131437       2.5645722028     o
 -5.6428710479      11.2857420958       2.5645722028     o
 -5.6428710479      16.9286131437       2.5645722028     o
```

3 CO on 2 × 2 MgO surface ($\Theta = 0.75$)

```
$cell
11.285742096   11.285742096  90
$coord
-14.1071783403      19.7500479471       7.12154955       c
 -8.4643072924      14.1071768992       7.12154955       c
-14.1071783403      14.1071768992       7.12154955       c
-14.1071783403      19.7500479471       9.277101446      o
 -8.4643072924      14.1071768992       9.277101446      o
```

```
-14.1071783403      14.1071768992       9.277101446      o
-14.1071783403      14.1071768992       2.4728236184    mg
-14.1071783403      19.7500479471       2.4728236184    mg
-11.2857420958      11.2857420958      -1.5186979106    mg
 -8.4643072924      14.1071768992       2.4728236184    mg
-11.2857420958      16.9286131437      -1.5186979106    mg
 -8.4643072924      19.7500479471       2.4728236184    mg
 -5.6428710479      11.2857420958      -1.5186979106    mg
 -5.6428710479      16.9286131437      -1.5186979106    mg
-14.1071783403      14.1071768992      -1.5186979106     o
-14.1071783403      19.7500479471      -1.5186979106     o
 -8.4643072924      14.1071768992      -1.5186979106     o
-11.2857420958      11.2857420958       2.5645722028     o
 -8.4643072924      19.7500479471      -1.5186979106     o
-11.2857420958      16.9286131437       2.5645722028     o
 -5.6428710479      11.2857420958       2.5645722028     o
 -5.6428710479      16.9286131437       2.5645722028     o
```

4 CO on 2×2 MgO surface ($\Theta = 1.00$)

```
$cell
11.285742096   11.285742096  90
$coord
-14.1071783403      19.7500479471       7.1215495 5      c
 -8.4643072924      14.1071768992       7.1215495        c
-14.1071783403      14.1071768992       7.12154955       c
-8.4643072924       19.7500479471       7.12154955       c
-14.1071783403      19.7500479471       9.277101446      o
 -8.4643072924      14.1071768992       9.277101446      o
-14.1071783403      14.1071768992       9.277101446      o
-8.4643072924       19.7500479471       9.277101446      o
-14.1071783403      14.1071768992       2.4728236184    mg
-14.1071783403      19.7500479471       2.4728236184    mg
-11.2857420958      11.2857420958      -1.5186979106    mg
 -8.4643072924      14.1071768992       2.4728236184    mg
-11.2857420958      16.9286131437      -1.5186979106    mg
 -8.4643072924      19.7500479471       2.4728236184    mg
 -5.6428710479      11.2857420958      -1.5186979106    mg
 -5.6428710479      16.9286131437      -1.5186979106    mg
-14.1071783403      14.1071768992      -1.5186979106     o
-14.1071783403      19.7500479471      -1.5186979106     o
 -8.4643072924      14.1071768992      -1.5186979106     o
-11.2857420958      11.2857420958       2.5645722028     o
 -8.4643072924      19.7500479471      -1.5186979106     o
-11.2857420958      16.9286131437       2.5645722028     o
 -5.6428710479      11.2857420958       2.5645722028     o
 -5.6428710479      16.9286131437       2.5645722028     o
```

4 CO on 4×4 MgO surface ($\Theta = 0.25$)

```
$cell
22.571484192 22.571484192  90
$coord
14.1071783403      -14.1071783403       7.1215495500     c
 -2.8214362445      -2.8214362445       7.1215495500     c
 -2.8214362445     -14.1071783403       7.1215495500     c
-14.1071783403      -2.8214362445       7.1215495500     c
-14.1071783403     -14.1071783403       9.2771014460     o
 -2.8214362445      -2.8214362445       9.2771014460     o
```

```
 -2.8214362445    -14.1071783403      9.2771014460     o
-14.1071783403     -2.8214362445      9.2771014460     o
-16.9286131437    -16.9286131437     -1.5186979106    mg
-14.1071783403    -14.1071783403      2.4728236184    mg
-16.9286131437    -11.2857420958     -1.5186979106    mg
-14.1071783403     -8.4643072924      2.4728236184    mg
-16.9286131437     -5.6428710479     -1.5186979106    mg
-14.1071783403     -2.8214362445      2.4728236184    mg
-16.9286131437      0.0000000000     -1.5186979106    mg
-14.1071783403      2.8214348034      2.4728236184    mg
-11.2857420958    -16.9286131437     -1.5186979106    mg
 -8.4643072924    -14.1071783403      2.4728236184    mg
-11.2857420958    -11.2857420958     -1.5186979106    mg
 -8.4643072924     -8.4643072924      2.4728236184    mg
-11.2857420958     -5.6428710479     -1.5186979106    mg
 -8.4643072924     -2.8214362445      2.4728236184    mg
-11.2857420958      0.0000000000     -1.5186979106    mg
 -8.4643072924      2.8214348034      2.4728236184    mg
 -5.6428710479    -16.9286131437     -1.5186979106    mg
 -2.8214362445    -14.1071783403      2.4728236184    mg
 -5.6428710479    -11.2857420958     -1.5186979106    mg
 -2.8214362445     -8.4643072924      2.4728236184    mg
 -5.6428710479     -5.6428710479     -1.5186979106    mg
 -2.8214362445     -2.8214362445      2.4728236184    mg
 -5.6428710479      0.0000000000     -1.5186979106    mg
 -2.8214362445      2.8214348034      2.4728236184    mg
  0.0000000000    -16.9286131437     -1.5186979106    mg
  2.8214348034    -14.1071783403      2.4728236184    mg
  0.0000000000    -11.2857420958     -1.5186979106    mg
  2.8214348034     -8.4643072924      2.4728236184    mg
  0.0000000000     -5.6428710479     -1.5186979106    mg
  2.8214348034     -2.8214362445      2.4728236184    mg
  0.0000000000      0.0000000000     -1.5186979106    mg
  2.8214348034      2.8214348034      2.4728236184    mg
-14.1071783403    -14.1071783403     -1.5186979106     o
-16.9286131437    -16.9286131437      2.5645722028     o
-14.1071783403     -8.4643072924     -1.5186979106     o
-16.9286131437    -11.2857420958      2.5645722028     o
-14.1071783403     -2.8214362445     -1.5186979106     o
-16.9286131437     -5.6428710479      2.5645722028     o
-14.1071783403      2.8214348034     -1.5186979106     o
-16.9286131437      0.0000000000      2.5645722028     o
 -8.4643072924    -14.1071783403     -1.5186979106     o
-11.2857420958    -16.9286131437      2.5645722028     o
 -8.4643072924     -8.4643072924     -1.5186979106     o
-11.2857420958    -11.2857420958      2.5645722028     o
 -8.4643072924     -2.8214362445     -1.5186979106     o
-11.2857420958     -5.6428710479      2.5645722028     o
 -8.4643072924      2.8214348034     -1.5186979106     o
-11.2857420958      0.0000000000      2.5645722028     o
 -2.8214362445    -14.1071783403     -1.5186979106     o
 -5.6428710479    -16.9286131437      2.5645722028     o
 -2.8214362445     -8.4643072924     -1.5186979106     o
 -5.6428710479    -11.2857420958      2.5645722028     o
 -2.8214362445     -2.8214362445     -1.5186979106     o
 -5.6428710479     -5.6428710479      2.5645722028     o
 -2.8214362445      2.8214348034     -1.5186979106     o
 -5.6428710479      0.0000000000      2.5645722028     o
  2.8214348034    -14.1071783403     -1.5186979106     o
  0.0000000000    -16.9286131437      2.5645722028     o
```

```
  2.8214348034      -8.4643072924      -1.5186979106     o
  0.0000000000     -11.2857420958       2.5645722028     o
  2.8214348034      -2.8214362445      -1.5186979106     o
  0.0000000000      -5.6428710479       2.5645722028     o
  2.8214348034       2.8214348034      -1.5186979106     o
  0.0000000000       0.0000000000       2.5645722028     o
```

8 CO on 4×4 MgO surface ($\Theta = 0.50$)

```
$cell
22.571484192 22.571484192  90
$coord
-14.1071783403     -14.1071783403       7.12154955       c
-8.4643072924       -8.4643072924       7.12154955       c
-2.8214362445       -2.8214362445       7.12154955       c
 2.8214348034        2.8214348034       7.12154955       c
-2.8214362445      -14.1071783403       7.12154955       c
2.8214348034        -8.4643072924       7.12154955       c
-14.1071783403      -2.8214362445       7.12154955       c
-8.4643072924        2.8214348034       7.12154955       c
-14.1071783403     -14.1071783403       9.277101446      o
-8.4643072924       -8.4643072924       9.277101446      o
-2.8214362445       -2.8214362445       9.277101446      o
 2.8214348034        2.8214348034       9.277101446      o
-2.8214362445      -14.1071783403       9.277101446      o
2.8214348034        -8.4643072924       9.277101446      o
-14.1071783403      -2.8214362445       9.277101446      o
-8.4643072924        2.8214348034       9.277101446      o
-16.9286131437     -16.9286131437      -1.5186979106    mg
-14.1071783403     -14.1071783403       2.4728236184    mg
-16.9286131437     -11.2857420958      -1.5186979106    mg
-14.1071783403      -8.4643072924       2.4728236184    mg
-16.9286131437      -5.6428710479      -1.5186979106    mg
-14.1071783403      -2.8214362445       2.4728236184    mg
-16.9286131437       0.0000000000      -1.5186979106    mg
-14.1071783403       2.8214348034       2.4728236184    mg
-11.2857420958     -16.9286131437      -1.5186979106    mg
 -8.4643072924     -14.1071783403       2.4728236184    mg
-11.2857420958     -11.2857420958      -1.5186979106    mg
 -8.4643072924      -8.4643072924       2.4728236184    mg
-11.2857420958      -5.6428710479      -1.5186979106    mg
 -8.4643072924      -2.8214362445       2.4728236184    mg
-11.2857420958       0.0000000000      -1.5186979106    mg
 -8.4643072924       2.8214348034       2.4728236184    mg
 -5.6428710479     -16.9286131437      -1.5186979106    mg
 -2.8214362445     -14.1071783403       2.4728236184    mg
 -5.6428710479     -11.2857420958      -1.5186979106    mg
 -2.8214362445      -8.4643072924       2.4728236184    mg
 -5.6428710479      -5.6428710479      -1.5186979106    mg
 -2.8214362445      -2.8214362445       2.4728236184    mg
 -5.6428710479       0.0000000000      -1.5186979106    mg
 -2.8214362445       2.8214348034       2.4728236184    mg
  0.0000000000     -16.9286131437      -1.5186979106    mg
  2.8214348034     -14.1071783403       2.4728236184    mg
  0.0000000000     -11.2857420958      -1.5186979106    mg
  2.8214348034      -8.4643072924       2.4728236184    mg
  0.0000000000      -5.6428710479      -1.5186979106    mg
  2.8214348034      -2.8214362445       2.4728236184    mg
  0.0000000000       0.0000000000      -1.5186979106    mg
  2.8214348034       2.8214348034       2.4728236184    mg
```

```
-14.1071783403    -14.1071783403    -1.5186979106    o
-16.9286131437    -16.9286131437     2.5645722028    o
-14.1071783403     -8.4643072924    -1.5186979106    o
-16.9286131437    -11.2857420958     2.5645722028    o
-14.1071783403     -2.8214362445    -1.5186979106    o
-16.9286131437     -5.6428710479     2.5645722028    o
-14.1071783403      2.8214348034    -1.5186979106    o
-16.9286131437      0.0000000000     2.5645722028    o
 -8.4643072924    -14.1071783403    -1.5186979106    o
-11.2857420958    -16.9286131437     2.5645722028    o
 -8.4643072924     -8.4643072924    -1.5186979106    o
-11.2857420958    -11.2857420958     2.5645722028    o
 -8.4643072924     -2.8214362445    -1.5186979106    o
-11.2857420958     -5.6428710479     2.5645722028    o
 -8.4643072924      2.8214348034    -1.5186979106    o
-11.2857420958      0.0000000000     2.5645722028    o
 -2.8214362445    -14.1071783403    -1.5186979106    o
 -5.6428710479    -16.9286131437     2.5645722028    o
 -2.8214362445     -8.4643072924    -1.5186979106    o
 -5.6428710479    -11.2857420958     2.5645722028    o
 -2.8214362445     -2.8214362445    -1.5186979106    o
 -5.6428710479     -5.6428710479     2.5645722028    o
 -2.8214362445      2.8214348034    -1.5186979106    o
 -5.6428710479      0.0000000000     2.5645722028    o
  2.8214348034    -14.1071783403    -1.5186979106    o
  0.0000000000    -16.9286131437     2.5645722028    o
  2.8214348034     -8.4643072924    -1.5186979106    o
  0.0000000000    -11.2857420958     2.5645722028    o
  2.8214348034     -2.8214362445    -1.5186979106    o
  0.0000000000     -5.6428710479     2.5645722028    o
  2.8214348034      2.8214348034    -1.5186979106    o
  0.0000000000      0.0000000000     2.5645722028    o
```

S2 Basis sets

Orbital Basis Sets

'DZ' Basis Set

```
# c
*
   8  s
  6665.0000000      0.69200000000E-03
  1000.0000000      0.53290000000E-02
  228.00000000      0.27077000000E-01
  64.710000000      0.10171800000
  21.060000000      0.27474000000
  7.4950000000      0.44856400000
  2.7970000000      0.28507400000
 0.52150000000      0.15204000000E-01
   8  s
  6665.0000000     -0.14600000000E-03
  1000.0000000     -0.11540000000E-02
  228.00000000     -0.57250000000E-02
  64.710000000     -0.23312000000E-01
  21.060000000     -0.63955000000E-01
  7.4950000000     -0.14998100000
  2.7970000000     -0.12726200000
 0.52150000000      0.54452900000
   1  s
 0.15960000000       1.0000000000
   3  p
  9.4390000000      0.38109000000E-01
  2.0020000000      0.20948000000
 0.54560000000      0.50855700000
   1  p
 0.15170000000       1.0000000000
   1  d
 0.55000000000       1.0000000000
*
# o
*
   8  s
  11720.000000      0.71000000000E-03
  1759.0000000      0.54700000000E-02
  400.80000000      0.27837000000E-01
  113.70000000      0.10480000000
  37.030000000      0.28306200000
  13.270000000      0.44871900000
  5.0250000000      0.27095200000
  1.0130000000      0.15458000000E-01
   8  s
  11720.000000     -0.16000000000E-03
  1759.0000000     -0.12630000000E-02
  400.80000000     -0.62670000000E-02
  113.70000000     -0.25716000000E-01
  37.030000000     -0.70924000000E-01
  13.270000000     -0.16541100000
  5.0250000000     -0.11695500000
  1.0130000000      0.55736800000
   1  s
 0.30230000000       1.0000000000
   3  p
```

```
  17.700000000       0.43018000000E-01
  3.8540000000       0.22891300000
  1.0460000000       0.50872800000
   1   p
 0.27530000000        1.0000000000
   1   d
  1.1850000000        1.0000000000
*
# mg
*
   5   s
  4953.8339196      -0.57778967498E-02
  745.18044154      -0.43124761082E-01
  169.21604972      -0.19268216987
  47.300672019      -0.48641439116
  14.461336973      -0.42550894077
   3   s
  24.768174789       0.87956969984E-01
  2.4940945349      -0.55165058128
  0.87807584530     -0.53443294833
   1   s
  0.34506887000      1.0000000000
   1   s
  0.15005399000      1.0000000000
   1   s
  2.911600E+00       1.000000E+00
   5   p
  98.053010494      -0.14480564601E-01
  22.586932277      -0.95495750787E-01
  6.8391509842      -0.30787672651
  2.2332843818      -0.49936292886
  0.71606599390     -0.31503476213
   1   p
  0.24692323000      1.0000000000
   1   p
  6.031000E+00       1.000000E+00
   1   d
  1.932000D-01       1.0000000
   1   d
  1.370200E+00       1.000000E+00
*
```

'TZ' Basis Set

```
# c
*
   8   s
  8236.0000000       0.53100000000E-03
  1235.0000000       0.41080000000E-02
  280.80000000       0.21087000000E-01
  79.270000000       0.81853000000E-01
  25.590000000       0.23481700000
  8.9970000000       0.43440100000
  3.3190000000       0.34612900000
 0.36430000000      -0.89830000000E-02
   8   s
  8236.0000000      -0.11300000000E-03
  1235.0000000      -0.87800000000E-03
  280.80000000      -0.45400000000E-02
  79.270000000      -0.18133000000E-01
```

```
  25.590000000      -0.55760000000E-01
  8.9970000000      -0.12689500000
  3.3190000000      -0.17035200000
 0.36430000000       0.59868400000
   1  s
 0.90590000000        1.0000000000
   1  s
 0.12850000000        1.0000000000
   3  p
  18.710000000       0.14031000000E-01
  4.1330000000       0.86866000000E-01
  1.2000000000       0.29021600000
   1  p
 0.38270000000        1.0000000000
   1  p
 0.12090000000        1.0000000000
   1  d
  1.0970000000        1.0000000000
   1  d
 0.31800000000        1.0000000000
   1  f
 0.76100000000        1.0000000000
*
# o
*
   8  s
  15330.000000       0.50800000000E-03
  2299.0000000       0.39290000000E-02
  522.40000000       0.20243000000E-01
  147.30000000       0.79181000000E-01
  47.550000000       0.23068700000
  16.760000000       0.43311800000
  6.2070000000       0.35026000000
 0.68820000000      -0.81540000000E-02
   8  s
  15330.000000      -0.11500000000E-03
  2299.0000000      -0.89500000000E-03
  522.40000000      -0.46360000000E-02
  147.30000000      -0.18724000000E-01
  47.550000000      -0.58463000000E-01
  16.760000000      -0.13646300000
  6.2070000000      -0.17574000000
 0.68820000000       0.60341800000
   1  s
  1.7520000000        1.0000000000
   1  s
 0.23840000000        1.0000000000
   3  p
  34.460000000       0.15928000000E-01
  7.7490000000       0.99740000000E-01
  2.2800000000       0.31049200000
   1  p
 0.71560000000        1.0000000000
   1  p
 0.21400000000        1.0000000000
   1  d
  2.3140000000        1.0000000000
   1  d
 0.64500000000        1.0000000000
   1  f
```

```
  1.4280000000        1.0000000000
*
# mg
*
   7  s
  31438.3495550       0.00060912311326
  4715.51533540       0.00470661964650
  1073.16292470       0.02413582065700
  303.572387680       0.09362895983400
  98.6262510420       0.26646742093000
  34.9438084170       0.47890929917000
  12.8597851990       0.33698490286000
   3  s
  64.8769130040       0.01918088930700
  19.7255207770       0.09091370439200
  2.89518043390      -0.39563756125000
   2  s
  1.19604547100        1.68276033730000
  0.54329451156        0.52141091954000
   1  s
  0.83471188300        1.00000000000000
   1  s
  0.14506887000        1.00000000000000
   1  s
  2.587700E+01         1.000000E+00
   1  s
  3.040200E+00         1.000000E+00
   5  p
  179.871896120       0.00537995490180
  42.1200693760       0.03931801409800
  13.1205030320       0.15740129476000
  4.62575036090       0.35919094128000
  1.66952110160       0.45533379310000
   1  p
  0.56631001000        1.00000000000000
   1  p
  0.18813966000        1.00000000000000
   1  p
  1.705300E+01         1.000000E+00
   1  p
  3.954100E+00         1.000000E+00
   1  d
  0.126000000          1.00000000
   1  d
  0.294000000          1.00000000
   1  d
  4.550700E+00         1.000000E+00
   1  d
  1.105100E+00         1.000000E+00
   1  f
  0.252000000          1.00000000
   1  f
  1.298500E+00         1.000000E+00
```

Auxiliary Basis Set

Standard Basis Set

The standard basis sets[2] are used for density fitting for non-ghost atoms, as mentioned in the main text.

'Dummy' Basis Set

These basis sets are used for density fitting for ghost atoms.

```
c dummy
# c
*
   1  s
  100000000000000      1.0000000000
*
mg dummy
# mg
*
   1  s
  100000000000000      1.0000000000
*
o dummy
# o
*
   1  s
  100000000000000      1.0000000000
*
```

S3 Further results

Dilute coverage results

Table S1 Hartree–Fock and MP2 correlation energies for each system involved in evaluating E_{int}, comparing supercell sizes of 3×3 and 5×5. All energies are given in Hartrees, unless stated. Calculations employed the $2 \cdot 2$ surface slab unit cell, using the modified 'TZ' basis set. This data was used for Table I, Table II and Figure 3 of the main text.

System	HF		$E_{\text{corr}}(\mathcal{T}_{\text{PNO}} = 10^{-7})$		$E_{\text{corr}}(\mathcal{T}_{\text{PNO}} = 10^{-8})$		CPS	
k_{super}	3^2	5^2	3^2	5^2	3^2	5^2	3^2	5^2
[MgOCO]	-2310.10796	-2310.10796	-4.14200	-4.14224	-4.14370	-4.14412	-4.14449	-4.14499
[MgO, $\overline{\text{CO}}$]	-2197.33009	-2197.33009	-3.77718	-3.77739	-3.77861	-3.77898	-3.77927	-3.77972
[CO, $\overline{\text{MgO}}$]	-112.77868	-112.77868	-0.35807	-0.35807	-0.35814	-0.35814	-0.35817	-0.35817
[CO]	-112.77842	-112.77842	-0.35712	-0.35712	-0.35714	-0.35714	-0.35715	-0.35715
[CO, $\overline{\text{CO}}$]	-112.77855	-112.77855	-0.35685	-0.35685	-0.35687	-0.35687	-0.35688	-0.35688
E_{int}	0.00094	0.00094	-0.00702	-0.00705	-0.00723	-0.00727	-0.00732	-0.00738
E_{int}(kJ mol^{-1})	2.47	2.47	-18.42	-18.51	-18.97	-19.11	-19.23	-19.39

Table S2 Hartree–Fock and MP2 correlation energies for each system involved in evaluating E_{int}, employing the $3 \cdot 3$ surface slab unit cell. All energies are given in Hartrees, unless stated. Calculations used a 3×3 supercell size, using the modified 'TZ' basis set. This data was used for Table II and Figure 3 of the main text.

System	HF	$E_{\text{corr}}(\mathcal{T}_{\text{PNO}} = 10^{-7})$	$E_{\text{corr}}(\mathcal{T}_{\text{PNO}} = 10^{-8})$	CPS
[MgOCO]	-5056.76941	-8.86202	-8.86541	-8.86698
[MgO, $\overline{\text{CO}}$]	-4943.99143	-8.49714	-8.50039	-8.50189
[CO, $\overline{\text{MgO}}$]	-112.77878	-0.35784	-0.35791	-0.35794
[CO]	-112.77852	-0.35689	-0.35691	-0.35693
[CO, $\overline{\text{CO}}$]	-112.77855	-0.35685	-0.35687	-0.35688
E_{int}	0.00083	-0.00708	-0.00716	-0.00719
E_{int}(kJ mol^{-1})	2.17	-18.59	-18.79	-18.89

Table S3 Hartree–Fock and MP2 correlation energies for each system involved in evaluating E_{int}, employing the 4·4 surface slab unit cell. All energies are given in Hartrees, unless stated. Calculations used a 3×3 supercell size, using the modified 'TZ' basis set. This data was used for Table II and Figure 3 of the main text.

System	HF	$E_{\text{corr}}(\mathcal{T}_{\text{PNO}} = 10^{-7})$	$E_{\text{corr}}(\mathcal{T}_{\text{PNO}} = 10^{-8})$	CPS
[MgOCO]	-8902.09505	-15.46972	-15.47562	-15.47835
[MgO, $\overline{\text{CO}}$]	-8789.31697	-15.10511	-15.11063	-15.11318
[CO, $\overline{\text{MgO}}$]	-112.77881	-0.35778	-0.35785	-0.35789
[CO]	-112.77855	-0.35684	-0.35686	-0.35687
[CO, $\overline{\text{CO}}$]	-112.77856	-0.35684	-0.35686	-0.35687
E_{int}	0.00074	-0.00683	-0.00714	-0.00729
E_{int}(kJ mol^{-1})	1.94	-17.92	-18.75	-19.13

Dense coverage results

Table S4 Hartree–Fock and MP2 correlation energies for each system involved in evaluating E_{int}, employing the $2 \cdot 2$ surface slab unit cell, with 1 adsorbed CO ($\Theta = \frac{1}{4}$). All energies are given in Hartrees, unless stated. Calculations used a 3×3 supercell size, using the modified 'TZ' basis set. This data was used for Table III and Figure 5 of the main text.

System	HF	$E_{\text{corr}}(\mathcal{T}_{\text{PNO}} = 10^{-7})$	$E_{\text{corr}}(\mathcal{T}_{\text{PNO}} = 10^{-8})$	CPS
[MgOCO]	-2310.10724	-4.14239	-4.14407	-4.14484
$[\text{MgO}, \overline{\text{CO}}]$	-2197.33047	-3.77730	-3.77861	-3.77921
$[\text{CO}, \overline{\text{MgO}}]$	-112.77869	-0.35809	-0.35815	-0.35818
[CO]	-112.77842	-0.35712	-0.35714	-0.35715
$[\text{CO}, \overline{\text{CO}}]$	-112.77855	-0.35685	-0.35687	-0.35688
E_{int}	0.00204	-0.00728	-0.00758	-0.00772
$E_{\text{int}}(\text{kJ mol}^{-1})$	5.37	-19.11	-19.90	-20.28

Table S5 Hartree–Fock and MP2 correlation energies for each system involved in evaluating E_{int}, employing the $4 \cdot 4$ surface slab unit cell, with 4 adsorbed COs ($\Theta = \frac{1}{4}$). All energies are given in Hartrees, unless stated. Calculations used a 3×3 supercell size, using the modified 'TZ' basis set. This data was used for Table III of the main text.

System	HF	$E_{\text{corr}}(\mathcal{T}_{\text{PNO}} = 10^{-7})$	$E_{\text{corr}}(\mathcal{T}_{\text{PNO}} = 10^{-8})$	CPS
[MgOCO]	-9240.42897	-16.57109	-16.57776	-16.58085
$[\text{MgO}, \overline{\text{CO}}]$	-8789.32188	-15.11015	-15.11593	-15.11860
$[\text{CO}, \overline{\text{MgO}}]$	-451.11475	-1.43239	-1.43266	-1.43278
[CO]	-451.11367	-1.42849	-1.42858	-1.42863
$[\text{CO}, \overline{\text{CO}}]$	-112.77855	-0.35685	-0.35687	-0.35688
E_{int}	0.00204	-0.00741	-0.00757	-0.00764
$E_{\text{int}}(\text{kJ mol}^{-1})$	5.36	-19.46	-19.88	-20.07

Table S6 Hartree–Fock and MP2 correlation energies for each system involved in evaluating E_{int}, employing the $2\cdot2$ surface slab unit cell, with 2 adsorbed COs ($\Theta = \frac{1}{2}$). All energies are given in Hartrees, unless stated. Calculations used a 3×3 supercell size, using the modified 'TZ' basis set. This data was used for Table III and Figure 5 of the main text.

System	HF	$E_{\mathrm{corr}}(\mathcal{T}_{\mathrm{PNO}} = 10^{-7})$	$E_{\mathrm{corr}}(\mathcal{T}_{\mathrm{PNO}} = 10^{-8})$	CPS
[MgOCO]	-2422.88403	-4.51129	-4.51323	-4.51412
$[\mathrm{MgO}, \overline{\mathrm{CO}}]$	-2197.33174	-3.77881	-3.78012	-3.78079
$[\mathrm{CO}, \overline{\mathrm{MgO}}]$	-225.55652	-0.71853	-0.71871	-0.71880
[CO]	-225.55602	-0.71658	-0.71669	-0.71674
$[\mathrm{CO}, \overline{\mathrm{CO}}]$	-112.77869	-0.35694	-0.35696	-0.35697
E_{int}	0.00279	-0.00833	-0.00856	-0.00866
$E_{\mathrm{int}}(\mathrm{kJ\ mol^{-1}})$	7.32	-21.86	-22.47	-22.75

Table S7 Hartree–Fock and MP2 correlation energies for each system involved in evaluating E_{int}, employing the $4\cdot4$ surface slab unit cell, with 8 adsorbed COs ($\Theta = \frac{1}{2}$). All energies are given in Hartrees, unless stated. Calculations used a 3×3 supercell size, using the modified 'TZ' basis set. This data was used for Table III of the main text.

System	HF	$E_{\mathrm{corr}}(\mathcal{T}_{\mathrm{PNO}} = 10^{-7})$	$E_{\mathrm{corr}}(\mathcal{T}_{\mathrm{PNO}} = 10^{-8})$	CPS
[MgOCO]	-9691.53612	-18.04651	-18.05435	-18.05798
$[\mathrm{MgO}, \overline{\mathrm{CO}}]$	-8789.32695	-15.11616	-15.12220	-15.12500
$[\mathrm{CO}, \overline{\mathrm{MgO}}]$	-902.22610	-2.87418	-2.87498	-2.87534
[CO]	-902.22410	-2.86639	-2.86685	-2.86706
$[\mathrm{CO}, \overline{\mathrm{CO}}]$	-112.77869	-0.35694	-0.35696	-0.35697
E_{int}	0.00279	-0.00838	-0.00854	-0.00861
$E_{\mathrm{int}}(\mathrm{kJ\ mol^{-1}})$	7.32	-22.01	-22.42	-22.62

Table S8 Hartree–Fock and MP2 correlation energies for each system involved in evaluating E_{int}, employing the $2\cdot2$ surface slab unit cell, with 3 adsorbed COs ($\Theta = \frac{3}{4}$). All energies are given in Hartrees, unless stated. Calculations used a 3×3 supercell size, using the modified 'TZ' basis set. This data was used for Figure 5 of the main text.

System	HF	$E_{\mathrm{corr}}(\mathcal{T}_{\mathrm{PNO}} = 10^{-7})$	$E_{\mathrm{corr}}(\mathcal{T}_{\mathrm{PNO}} = 10^{-8})$	CPS
[MgOCO]	-2535.64209	-4.89141	-4.89375	-4.89483
$[\mathrm{MgO}, \overline{\mathrm{CO}}]$	-2197.33217	-3.77989	-3.78129	-3.78194
$[\mathrm{CO}, \overline{\mathrm{MgO}}]$	-338.31405	-1.09127	-1.09163	-1.09180
[CO]	-338.31334	-1.08800	-1.08825	-1.08837
$[\mathrm{CO}, \overline{\mathrm{CO}}]$	-112.77883	-0.35723	-0.35726	-0.35728
E_{int}	0.00910	-0.01219	-0.01243	-0.01254
$E_{\mathrm{int}}(\mathrm{kJ\ mol^{-1}})$	23.88	-32.00	-32.64	-32.93

Table S9 Hartree–Fock and MP2 correlation energies for each system involved in evaluating E_{int}, employing the $2\cdot2$ surface slab unit cell, with 4 adsorbed COs ($\Theta = 1$). All energies are given in Hartrees, unless stated. Calculations used a 3×3 supercell size, using the modified 'TZ' basis set. This data was used for Figure 5 of the main text.

System	HF	$E_{\mathrm{corr}}(\mathcal{T}_{\mathrm{PNO}} = 10^{-7})$	$E_{\mathrm{corr}}(\mathcal{T}_{\mathrm{PNO}} = 10^{-8})$	CPS
[MgOCO]	-2648.39932	-5.27365	-5.27588	-5.27691
$[\mathrm{MgO}, \overline{\mathrm{CO}}]$	-2197.33265	-3.78105	-3.78247	-3.78312
$[\mathrm{CO}, \overline{\mathrm{MgO}}]$	-451.071166	-1.46611	-1.46677	-1.46707
[CO]	-451.07015	-1.46138	-1.46183	-1.46203
$[\mathrm{CO}, \overline{\mathrm{CO}}]$	-112.77900	-0.35754	-0.35759	-0.35761
E_{int}	0.01258	-0.01442	-0.01453	-0.01458
$E_{\mathrm{int}}(\mathrm{kJ\ mol^{-1}})$	33.04	-37.87	-38.15	-38.28

References

[1] H.-Z. Ye and T. C. Berkelbach, "Adsorption and vibrational spectroscopy of CO on the surface of MgO from periodic local coupled-cluster theory", *Faraday Discuss.* **254**, 628–640 (2024).
[2] F. Weigend, "Accurate Coulomb-fitting basis sets for H to Rn", *Phys. Chem. Chem. Phys.* **8**, 1057–1065 (2006).
